# Dissociative Identity Disorder from a Palestinian Perspective: A Case Report

**DOI:** 10.1192/j.eurpsy.2024.1085

**Published:** 2024-08-27

**Authors:** N. Marzouqa, S. Saadeh

**Affiliations:** Psychiatry, Dr Kamal Psychiatric Hospital, Bethlehem, Palestinian, State of

## Abstract

**Introduction:**

Dissociative Identity Disorder (DID) is the presence of two or more distinct personality states within an individual. It is a rare dissociative disorder where usually self-non-integration arises as a response to significant stress. As is the case for many other psychiatric disorders, the diagnosis and management of DID is highly dependent on cultural variables and contexts.

**Objectives:**

To present a case of DID in Palestine in which the diagnosis was dependent on noticing minor changes in the patient’s dialect at different times. To highlight the importance of understanding each patient’s environment, values, and culture when assessing DID to avoid under- or over-diagnoses.

**Methods:**

A case report in which we present a case of a 17-year-old Palestinian girl who suffered from three months of general fatigue, restlessness, poor coordination, and peripheral numbness. She was seen by several doctors who excluded organic causes and several spiritual healers without benefit. This caused severe deterioration of social and academic functioning. The family noticed a change in her articulation and memory issues, so they presented her to a psychiatrist. Data was collected by interviewing the patient and her family weekly for a month. The psychiatrist noted that the patient has subtle differences in accent, and directed the family to record any change in tone of voice or articulation. The patient was found to have three different accents on top of her native one, representing a total of four personalities with no memory integrity among them. She was started on Escitalopram (gradually increased) and Alprazolam (gradually decreased).

**Results:**

The psychiatrist detected the theme of being “stuck” throughout the personalities, each in its own way, according to the context of their roots. It was revealed that the patient is engaged to a man she doesn’t approve of and has “no way out” due to the social and familial significance of this relationship. Family counseling and trauma-informed psychoeducation were done, where the patient’s choice was reaffirmed. This led to significant improvement in terms of mood, identity integration, and social functioning with complete resolution of split personalities in just short of one-and-a-half months.

**Conclusions:**

This case report asserts the importance of cultural understanding and sensitivity when assessing psychiatric patients. Symptoms, triggers, psychotherapy, and psychoeducation have a universal baseline, yet are highly culturally-dependent. Through this case study, we emphasize the importance of translating universal criteria into context-specific practices.

**Disclosure of Interest:**

None Declared

